# TISNet-Enhanced Fully Convolutional Network with Encoder-Decoder Structure for Tongue Image Segmentation in Traditional Chinese Medicine

**DOI:** 10.1155/2020/6029258

**Published:** 2020-08-07

**Authors:** Xiaodong Huang, Hui Zhang, Li Zhuo, Xiaoguang Li, Jing Zhang

**Affiliations:** ^1^Faculty of Information Technology, Beijing University of Technology, Beijing 100124, China; ^2^Beijing Key Laboratory of Computational Intelligence and Intelligent System, Beijing University of Technology, Beijing 100124, China; ^3^Henan University of Science and Technology, Luoyang 471000, China

## Abstract

Extracting the tongue body accurately from a digital tongue image is a challenge for automated tongue diagnoses, as the blurred edge of the tongue body, interference of pathological details, and the huge difference in the size and shape of the tongue. In this study, an automated tongue image segmentation method using enhanced fully convolutional network with encoder-decoder structure was presented. In the frame of the proposed network, the deep residual network was adopted as an encoder to obtain dense feature maps, and a Receptive Field Block was assembled behind the encoder. Receptive Field Block can capture adequate global contextual prior because of its structure of the multibranch convolution layers with varying kernels. Moreover, the Feature Pyramid Network was used as a decoder to fuse multiscale feature maps for gathering sufficient positional information to recover the clear contour of the tongue body. The quantitative evaluation of the segmentation results of 300 tongue images from the SIPL-tongue dataset showed that the average Hausdorff Distance, average Symmetric Mean Absolute Surface Distance, average Dice Similarity Coefficient, average precision, average sensitivity, and average specificity were 11.2963, 3.4737, 97.26%, 95.66%, 98.97%, and 98.68%, respectively. The proposed method achieved the best performance compared with the other four deep-learning-based segmentation methods (including SegNet, FCN, PSPNet, and DeepLab v3+). There were also similar results on the HIT-tongue dataset. The experimental results demonstrated that the proposed method can achieve accurate tongue image segmentation and meet the practical requirements of automated tongue diagnoses.

## 1. Introduction

In the field of complementary medicine, tongue diagnosis is the most active approach compared to other diagnostic methods such as palpation and pulse diagnosis [1]. Because of its simplicity, immediacy, and effectiveness, tongue diagnosis is widely used in Traditional Chinese Medicine (TCM), Japanese traditional herbal medicine, and Traditional Korean Medicine (TKM) [2].

**Figure 1:**
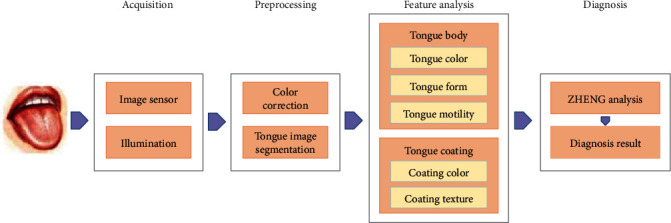
Schematic diagram of a typical computer-aided tongue diagnosis system.

Tongue diagnosis means that the doctors judge the status of a patient's internal organs by the visual information such as the color, form, substance, and coating of the tongue. The diagnostic results heavily rely on the doctor's experiences. Moreover, the inspecting circumstances, such as illumination, also affects the judgment of doctors. So, it is difficult for tongue diagnosis to obtain objective and standardized results. In the past decades, researchers have developed various types of computer-aided tongue diagnosis systems (CATDS) based on computer vision and machine learning to address these problems in many countries [3–6]. The schematic diagram of a typical CATDS is shown in Figure 1 [7]. The system is mainly composed of four ordered modules: tongue image acquisition, image preprocessing, feature analysis, and diagnosis. Plenty of research have already been carried out on each module.

**Figure 2:**
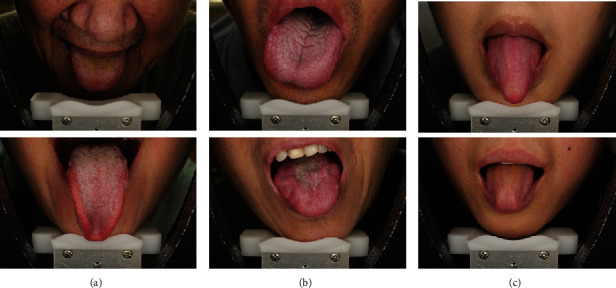
Difficult cases for automated tongue image segmentation. (a) Tongue bodies with different size and shape. (b) Severe fissures and teeth marks on the surface of a tongue body. (c) Similar color between tongue body and lips.

Generally, tongue images acquired by CATDSs include some nontongue regions, such as the face, teeth, and lips. However, the results of tongue diagnosis are only related to the tongue body according to the theory of traditional oriental medicine. So, it is an essential step to segment the tongue body from a tongue image in CATDSs. Furthermore, high accuracy is hugely demanded in tongue image segmentation because the accurate results of the tongue feature analysis mainly depend on the result of segmentation. This close relationship should be emphasized because pathological information may exist on any part of the tongue body, for example, teeth marks, which is one of the important tongue features with rich disease-related information, usually occur at the edge of the tongue body. For automated tongue image segmentation, there are still some factors that make it challenging to extract tongue body accurately and robustly. The specific cases are shown in Figure 2.

- Different shooting angle and circumstance and wide variations of patients in the shape and size of the tongue body
- The variation in tongue color, texture, and shape due to the pathology of the tongue
- A blurred edge of the tongue body caused by a similar color between the tongue body and the lips.

During the past decades, researchers have developed many methods to segment tongue body from digital tongue images effectively. Generally, these methods can be divided into two categories: methods based on traditional image processing technology and methods based on deep learning.

In the first category, some methods employed the techniques of the adaptive threshold, region growing and merging, and edge detection to segment the tongue body from tongue images [8–10]. However, these segmentation methods just took the low-level visual information (such as color, edge, and texture) into account and often failed to extract the tongue body from the surroundings completely. They ignored high-level information such as shape attribute, which is an indispensable factor for this segmentation task. Therefore, the other methods which introduce high-level information were proposed to achieve better performance. The active contour model (ACM) [11] was the most widely used method based on high-level information in the field of tongue image segmentation in the first category, which is also known as Snakes. According to the initialization methods and the strategy of curve evolution, there were many variants of the ACM-based algorithms. Concerning the initialization, Pang et al. [12] applied the bielliptical deformable template to obtain the initial evolving contour. Zuo et al. [13] used a polar edge detector to detect the initial boundary of the tongue body. Furthermore, Wu et al. [14] applied the watershed transformation to obtain the initial contour of the snake. Regarding the curve evolution, Yu et al. [15] extracted the tongue body by the addition of a color gradient to the gradient vector flow (GVF) snake. Shi et al. [16] applied double geodesic flow to extract tongue body based on the prior information of tongue shape and location. Later, Shi et al. [17] continued the work with the color control-geometric and gradient flow snake algorithm-enhanced curve velocity. However, the traditional methods still could not meet the demands of accuracy and robustness simultaneously, which are essential for the following automated analysis of the tongue features.

Since 2012, the convolutional neural network (CNN) has made significant progress in various fields of computer vision. Some researchers also tried to apply CNNs to tongue image segmentation [18–20]. In these studies, the methods based on deep learning could get better results than traditional methods. However, it was still difficult for these methods to get accurate segmentation results because of their simple network architecture, when they deal with similar cases shown in Figure 2.

After Shelhamer et al. [21] introduced the fully convolutional neural network to the field of image segmentation, various new models emerged and achieved remarkable results in the field of image segmentation. There were still several factors that affect the accuracy of segmentation results. The first one is related to the fact that the effective receptive field on high-level layers of CNN is much smaller than the theoretical one, especially on high-level layers [22], which may make many networks only “see” a small part of the entire image and be unable to integrate the sufficient contextual prior. Some methods employed spatial pyramid pooling to capture multiscale context to address the problem. DeepLab v3+ [23] used Atrous Spatial Pyramid Pooling (ASPP) to capture contextual information. In [24], ASPP was replaced by the Vortex Pooling Module, which can aggregate features around the target position more efficiently by assigning different attention. Pyramid scene parsing net [25] introduced the pyramid pooling module to contain information with different scales. All of the above works demonstrated outstanding performance. The second factor is caused by the inherent limitation of CNN. In the task of image classification, deeper networks with multiple down-sampling by max-pooling or convolution operation (stride > 1) have proven to be most successful. However, for image segmentation, higher layer feature maps usually contain more semantic meanings and yet lose more positional information inevitably, which may cause rough object boundaries. Meanwhile, clear and sharp object boundaries are critical in some applications. The researchers have proceeded in two directions to deal with this problem [25]. The first one was the structure prediction. In researches [26–28], the conditional random field (CRF) was adopted to improve the segmentation result. The other direction was to fuse feature maps from different intermediate layers in CNNs to predict the object boundaries better. The skip connection or encoder-decoder structure was the primary form of multiscale feature map fusion. DeepLab v3+ and RefineNet [29], which use the encoder-decoder structure, showed effectiveness in the field of image segmentation.

Inspired by these works, an automated tongue image segmentation network named TISNet was presented, which was based on the fully convolutional network with encoder-decoder structure. The ResNet101 [30] was adopted as an encoder to obtain dense feature maps, and the Receptive Field Block (RFB) [31] was assembled behind the encoder to integrate adequate global contextual prior. Moreover, a Feature Pyramid Network (FPN) [32] was used as a decoder to fuse multiscale feature maps to recover the clear and sharp boundary of the tongue body.

## 2. Methods

As mentioned above, for a well-performance segmentation network, it is significant to have a large receptive field for integrating adequate global contextual prior and maintain more positional information at the same time. In the TISNet, Receptive Field Block was introduced to integrate adequate global contextual prior, and Feature Pyramid Network was adopted to gather sufficient positional information to cover the contour of the tongue body. Next, the structures of RFB, FPN, and the architecture of TISNet were described in detail.

### 2.1. Receptive Field Block

Currently, in several state-of-art segmentation networks, a particular module is designed to obtain global contextual information for more accurate segmentation results [23–25, 33, 34]. However, these modules commonly set the receptive field of the same size on the feature map with a regular sampling grid, which may cause some loss in the robustness and discernibility of the feature. For example, the Atrous Spatial Pyramid Pooling (ASPP) module used in DeepLab v3+ is employed to capture the multiscale context. The ASPP module, which has several parallel branches of convolutional layers with different atrous rates, is applied on the top feature maps. Though the ASPP module can change the sampling distance from the center of feature maps, resulting features captured by it still have a uniform resolution and tend to be less distinctive than daisy-shaped ones. There are the same problems in Pyramid Pooling Module [25] and Vortex Pooling Module [34].

In the field of neuroscience, researchers find that the size of population Receptive Field (pRF) is a function of eccentricity in the retinotopic maps in the human visual system, and the size of pRF increases with eccentricity in spite of varying in the maps. This mechanism means that the region closer to the center of retinotopic maps is more important than others in distinguishing object [35].

**Figure 3:**
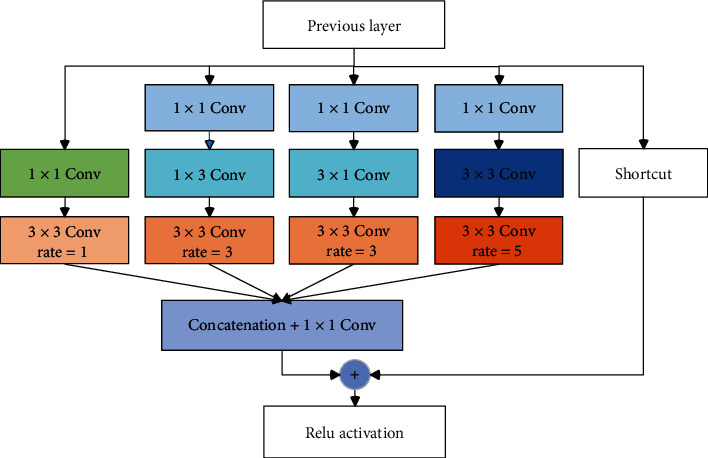
The structures of Receptive Field Block.

Inspired by the mechanism of pRF in the human visual system, a module named Receptive Field Block was introduced to strengthen global contextual prior information learned from CNN in this study, which is helpful for accurate image segmentation.  Figure 3 illustrates the details of the internal structure of RFB. The RFB includes a multibranch convolutional block and a shortcut connection. The convolutional block consists four branches, and each branch has a convolutional layer with a particular kernel size and a corresponding atrous convolutional layer with the same value in dilation rate. The functional relation between the kernel size and dilation rate is similar to that of the size and eccentricity of pRFs in the human visual system. The ordinary convolutional layer generates the multisize pRF, and the atrous convolutional layer simulates the human visual system to build the relationship between the size and eccentricity of pRF. Finally, all feature maps from the different branches are merged into one convolutional layer and pixel-wise added with the shortcut connection branch.

### 2.2. Feature Pyramid Network

Before pixel prediction has arisen, segmentation models generally adopt CNN models as a backbone to extract the feature maps. However, these CNN models transferred from the tasks of image classification usually lose more positional information due to CNN invariance, which causes that segmentation network cannot obtain accurate object boundaries. Therefore, for better performance, some of the new methods of image segmentation attend to use the different layers in a CNN [21, 36] or concatenate the feature maps from multiple layers before final mask predictions [37, 38]. Also, some methods associate the high-level semantic information with low-level feature maps though lateral/skip connections to recover the positional information [39, 40].

**Figure 4:**
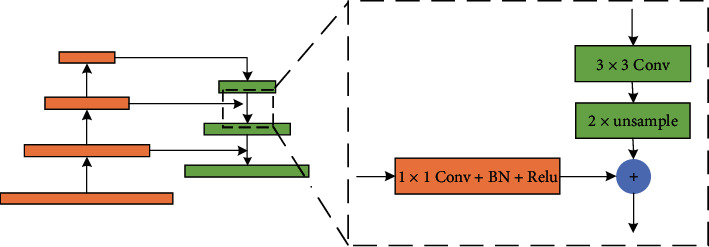
The structures of Receptive Field Block.

In this paper, the Feature Pyramid Network (FPN) was introduced to gather more positional information for obtaining fine object boundaries. Giving a single-scale image of arbitrary size, the FPN can generate a set of feature maps with proportionally sized at multilevel. As shown in Figure 4, the structure of FPN consists three components: a bottom-up pathway, a top-down pathway, and lateral connections. The details of FPN would be explained in the next section.

### 2.3. Tongue Image Segmentation Network

**Figure 5:**
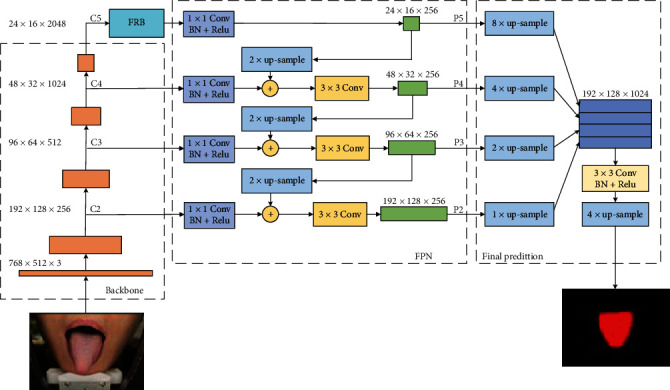
The architecture of the tongue image segmentation network.

As shown in Figure 5, the TISNet consists four parts: backbone module, RFB module, FPN module, and final prediction module. The ResNet101 is adopted as a backbone to extract the feature maps, and the feature maps output by each stage's last residual block are used. The feature maps of the last residual blocks of conv2, conv3, conv4, and conv5 are denoted as C2, C3, C4, and C5, which correspond to the strides of 4, 8, 16, and 32 pixels to the input image. The conv1 is not included because of the large requirement of memory.

Behind the backbone, the RFB module is applied to integrate adequate global contextual prior. The structure of the RFB module has been described in detail in Section 3.1. By four branches, the RFB module can cover the whole, half, and small portions of the image. The feature maps from all the branches are fused and then fed into the FPN. The details of the FPN is shown in the box of FPN. It fuses high-level semantic information with the middle- and low-level features by lateral connections in the top-down path. The coarser-resolution feature maps are up-sampled by a factor of 2 and then merged with the corresponding feature maps from the backbone by element-wise addition. Before merging, the feature maps from backbone go though a 1 × 1 convolutional layer to reduce channel dimensions. Then, a 3 × 3 convolution operation is applied to the merged map to reduce the aliasing effect of up-sampling before producing the final feature maps of this level. This process iterates until the highest resolution feature maps are generated. The set of final feature maps are denoted as P2, P3, P4, and P5, which correspond to C2, C3, C4, and C5, and have the same spatial sizes. Also, the number of channels of all final feature maps in each level is set to 256 for simplifying the computational complexity.

In the final prediction module, a strategy of fusion of multiscale feature maps is applied to recover the contour details instead of only using the feature map with the highest resolution (P2). The final set of feature maps P3, P4, and P5 is first bilinearly up-sampled to the same size of P2, and then all of the four feature maps are concatenated. After the concatenation, a 3 × 3 convolution is appended on the feature maps, followed by another simple bilinear up-sampled by a factor of 4 to obtain the final mask.

## 3. Experiments and Results

In this section, the performance of TISNet was evaluated on two tongue image datasets: HIT-tongue dataset and SIPL-tongue dataset. All experiments were conducted on the public platform PyTorch 0.4.1 on the Windows system. The system configuration is Intel (R) i5-7500 CPU @ 3.4GHz with 16 G memory and NVIDIA Titan XP graphics card.

### 3.1. Implementation Details

#### 3.1.1. Datasets

*(1)HIT-Tongue Dataset*. This dataset is an open tongue image dataset from the Harbin Institute of Technology. The dataset contains 300 RGB tongue images, and the corresponding manual masks as the ground truth. All the tongue images are acquired in a semienclosed environment under stable lighting conditions. The image size is 768 × 576.

*(2) SIPL-Tongue Dataset*. This dataset is constructed exclusively for automated tongue image analysis by ourselves. All the images are acquired by a self-designed acquisition device in a closed environment and carefully selected and labelled by the experts in Chinese Traditional Medicine from Beijing Xuanwu Hospital. In the SIPL-tongue dataset, there are 700 RGB tongue images and the corresponding well-labelled masks for tongue image segmentation. The image size is 1024 × 768.

#### 3.1.2. Data Augmentation

The research suggests that the number of training samples is critical to the performance of CNN. For a small-scale dataset, artificial data augmentation is a common method to generate enough training samples. Because of the limited scale of the tongue image dataset, the strategies, including random Horizontal Flip, random resize between 0.5 and 2, random rotation between -10 and 10 degrees, and random Gaussian blur, were adopted for data augmentation in this study.

#### 3.1.3. Training Parameters

In this study, the weights of pretrained Resnet101 were directly employed in the backbone module of the TISNet. Then TISNet was trained on the PASCAL VOC 2012 semantic segmentation benchmark [41] and was fine-tuned, respectively, on two tongue image datasets.  Table 1 shows the parameters used in the training step.

### 3.2. Evaluation Criteria

In this study, there are six metrics used to evaluate the performance of TISNet.  Table 2 gives a detailed description of the six metrics.

### 3.3. Experimental Results

To validate the effectiveness of the proposed method, a comparison of performance was made between our method and the other four deep-learning-based methods (including FCN, SegNet, PSPNet, and DeepLab v3+) on the HIT-tongue dataset and SIPL-tongue dataset. For a fair comparison, these four models have the same training hyperparameters as our model and follow the same training procedure during experiments. Among these methods, FCN is the first work that trains a fully convolutional network end-to-end for semantic segmentation and exceeds the state-of-the-art at that time. Many follow-up excellent works are based on FCN. SegNet is a model with the encoder-decoder structure for pixel-wise image segmentation and obtains a good balance between accuracy and requirement of memory. PSPNet embeds a pyramid pooling module into the scene parsing network to aggregate region-based context and achieves state-of-the-art performance on various datasets by exploiting the capability of global context information. DeepLab v3+ combines the advantages of spatial pyramid pooling module and encoder-decoder structure and obtains the best results on the PASCAL VOC 2012 semantic segmentation benchmark [41] and Cityscapes datasets [42].

#### 3.3.1. Experiments on HIT-Tongue Dataset

**Figure 6:**
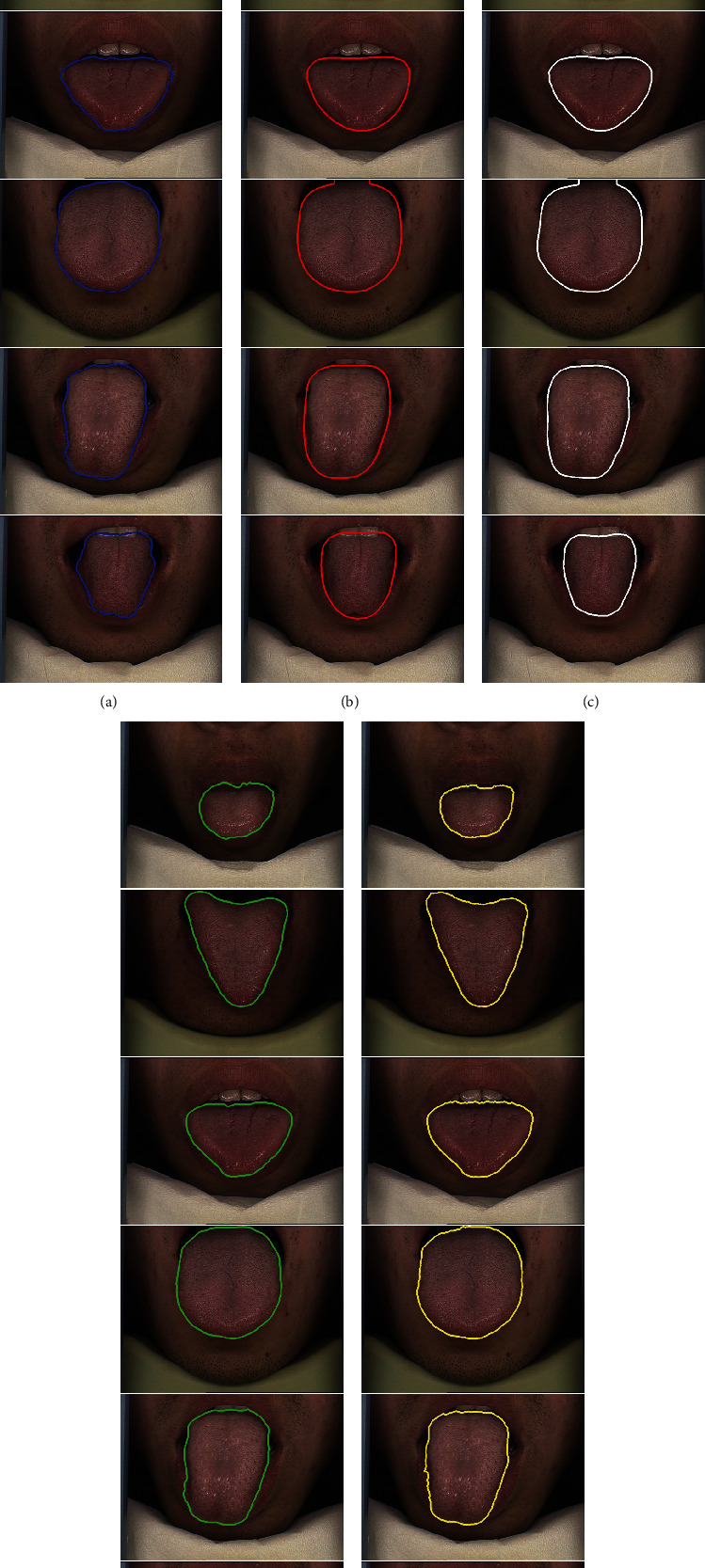
Segmentation results of tongue images from HIT-tongue dataset.

A comparison of the performance of five methods was conducted on the HIT-tongue dataset, and Table 3 shows the quantitative results. It can be seen that TISNet gets the best results in terms of DSC, HD, MSD, accuracy, and specificity. Also, Figure 6 exhibits the segmentation results of six typical cases from the HIT-tongue dataset. Rows 1 to 4 are the four cases in which the tongue body is with different size and shape. Rows 5 and 6 give two cases in which the color of the tongue body is similar to the lip, and the interference from the teeth is also visible. The segmentation results show that all deep models can locate the tongue body regardless of the difference in size and shape. Although FCN, SegNet, and PSPNet can obtain the smooth contour, the segmentation results are not accurate, usually accompanied by oversegmentation or undersegmentation, especially FCN. Instead, DeepLab v3+ and TISNet can retain more details and present clear and sharp contours.

#### 3.3.2. Experiments on SIPL-Tongue Dataset

**Figure 7:**
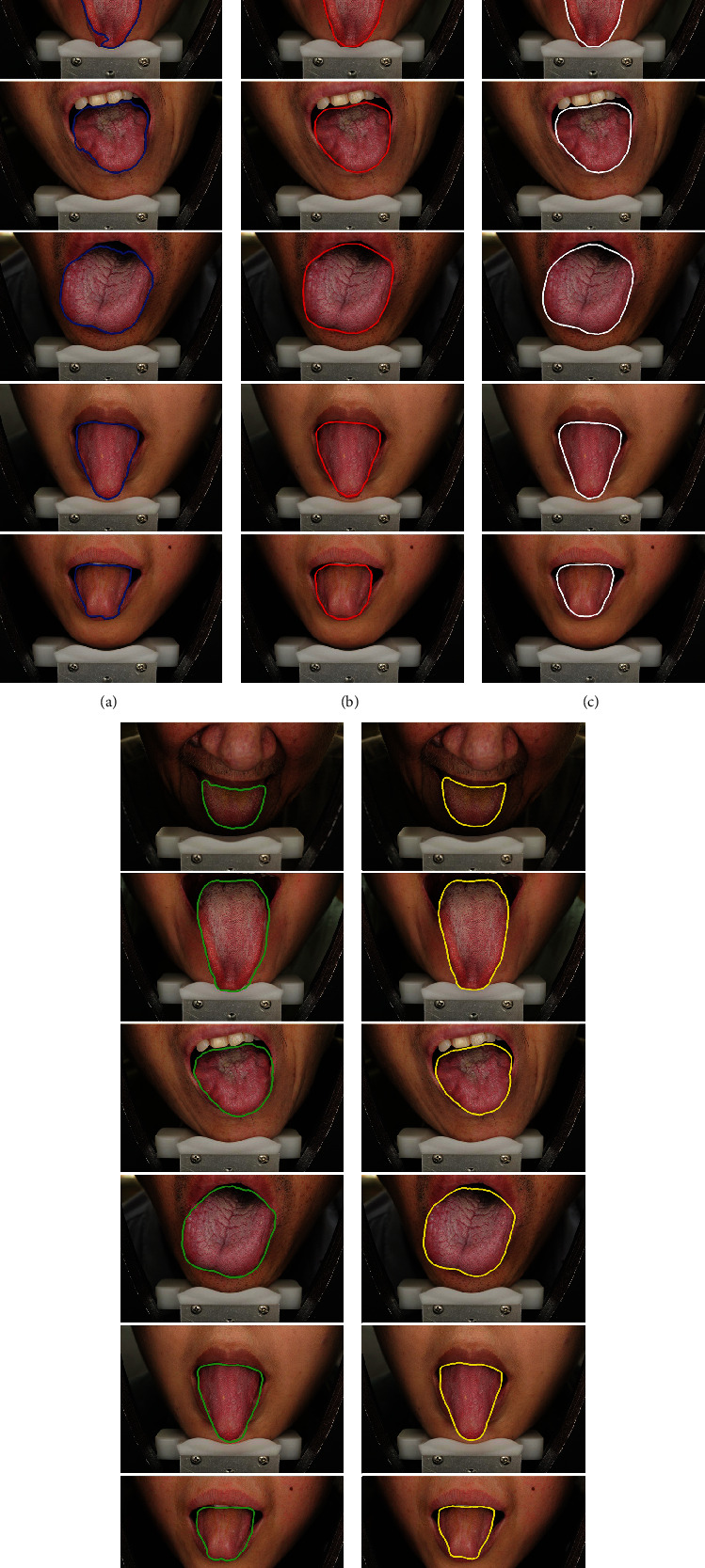
Segmentation results of tongue images from SIPL-tongue dataset.

Table 4 lists the quantitative results obtained by different methods. It can be seen that TISNet can get the best results on 5 out of 6 metrics and can also perform well in terms of sensitivity. TISNet has the lowest value in DSC, HD, and MSD along with small standard deviations, and this indicates that TISNet can obtain a more accurate contour of the tongue body. It also is confirmed by qualitative results shown in Figure 7, where the segmentation results of six cases from the SIPL-tongue dataset are illustrated. Rows 1 and 2 are two cases in which the tongue body is with different size and shape. Rows 3 and 4 show two cases in which there are severe fissures, tooth marks, and coating on the surface of the pathological tongue. Rows 5 and 6 are two cases in which the tongue body has blurred contour between the tongue body and the lip along with the interference from the teeth. The tongue images from the SIPL-tongue dataset are more diverse and complicated compared with the HIT-tongue dataset, which can test the performance of the model more effectively. From Figure 7, FCN and SegNet all fail to extract the tongue body in case 1, and there are oversegmentation or undersegmentation in the other five cases. Because of more complicated and powerful models, PSPNet and DeepLab v3+ get better results than FCN and SegNet, but they still cannot segment the tongue body accurately, and especially, there are obvious oversegmentation in case 5 and case 6. It means that PSPNet and DeepLab v3+ cannot deal with the problem of the blurred edge well. Instead, TISNet solves the problem better and gets the finest segmentation results, which principally benefits from the elaborately designed network structure.

#### 3.3.3. Ablation Study

In this study, RFB, FPN, and multiscale fusion strategy are employed to improve the performance of the segmentation network. The ablation study is conducted on the SIPL-tongue dataset to validate the effectiveness of these modules and strategies.  Table 5 shows the results of the ablation study. It should be noted that the “+” means adding a new module or strategy based on the last row instead of the baseline. The first row in Table 5 contains the results of the baseline. The baseline uses the Resnet101 pretrained on ImageNet as a backbone. A 3 × 3 convolution layer with channel dimension 2 is embedded on top of the backbone to classify the pixels, and then the coarse output is up-sampled to produce the final segmentation results. Because the size of the final outputs of the backbone is 1/32 of the input image, the scale of details in the final prediction is limited, which leads to the worst results. The second row in Table 5 shows the results when adding the RFB before the final prediction occurs. As we can see, the performance is overall improved, and especially, the HD is decreased from 17.0623 to 13.8587. Then, the FPN is embedded in the previous structure, which further improves the performance in all six metrics. Finally, after the multiscale fusion strategy is added, the best results are yielded and listed in the fourth row in Table 5. It is concluded that these modules and strategies reinforce the performance of the network for tongue image segmentation.

## 4. Discussion and Conclusion

In this study, a new tongue image segmentation method named TISNet was proposed, and it achieved the best performance on two tongue image datasets compared with the other four deep-learning-based methods. The qualitative and quantitative evaluation showed that the proposed method met the practical requirements of automated tongue image segmentation and could be embedded in computer-aided tongue diagnosis systems.

According to the experimental results, it can be concluded that the deep-learning-based methods are robust to the variation in the size and shape of the tongue body, as well as pathological details on the surface of the tongue. For the method of tongue image segmentation, the key point is to deal with the problem of the blurred edge of the tongue body to obtain a sharp and clear contour. An elaborately designed model is critical.

In this study, three factors make TISNet achieve the best performance. Firstly, the ResNet101 model is adopted as a backbone to extract the dense feature maps, which is also used in PSPNet and DeepLab v3+. The ResNet101 is more potent in feature extraction and representation than VGG16 [43] used as the backbone in FCN and SegNet. It is one of the reasons that TISNet, DeepLab v3+, and PSPNet can perform better than FCN and SegNet in total. Secondly, RFB is introduced behind the backbone to integrate adequate global contextual prior. Moreover, because the structure of the RFB simulates the pRF in the human vision system, the feature extracted by RFB is more distinctive than the ones obtained by PPM in PSPNet and by ASPP in DeepLab v3+. Finally, FPN is applied to produce a fine mask. FFN is a typical encoder-decoder structure and can gather sufficient positional information by fusing multiscale feature maps, and this positional information is much valuable for recovering the accurate contour of the tongue body.

In the future, other than applying the proposed method to a more number of clinical data, our research will focus on making the model light-weight and efficient to run it in mobile devices.

## Figures and Tables

**Table 1:** Training parameters of the proposed method.

Hyperparameters	Interpretation
Normalization	Mean centering and standard deviation normalization of the intensities were performed
Cropping	All the images were center-cropped to a 512 × 512 pixel size
Optimization	SGD optimizer with the base learning rate 0.01
Learning rate scheduling	The poly learning rate policy was used, where the current learning rate equals to the base one multiplying (1 − *n*/*N*)^power^
Batch size	4
Epoch number size	50
Momentum	0.9
Weight decay	0.0005

**Table 2:** Description of the evaluation metrics.

Metric name	Abbr.	Range	Interpretation	Category
Dice similarity coefficient	DSC	0–1	Similarity between masks	Overlap
Hausdorff distance	HD	>0	Longest Euclidean distance between mask contours (absolute error)	Distance
Symmetric mean absolute	MSD	>0	Mean Euclidean distance between mask contours (mean error)	Distance
Surface distance precision	PPV	0–1	Low values mean that the method tends to over segment	Statistical
Sensitivity	TPR	0–1	Low values mean that the method tends to under segment	Statistical
Specificity	TNR	0–1	Quality of segmented background	Statistical

**Table 3:** Evaluation of the segmentation results for 100 tongue images from the HIT-tongue dataset.

Method	DSC	HD (pixel)	MSD (pixel)	Precision	Sensitivity	Specificity
SegNet	0.9821 ± 0.0097	14.8461 ± 4.1231	3.0021 ± 2.0801	0.9814 ± 0.0153	0.9832 ± 0.0168	0.9893 ± 0.0082
FCN	0.9700 ± 0.0148	17.9651 ± 7.0000	4.8904 ± 2.8970	0.9646 ± 0.0246	0.9762 ± 0.0184	0.9792 ± 0.0143
PSPNet	0.9800 ± 0.0071	12.9046 ± 5.6969	3.2129 ± 1.0758	0.9806 ± 0.0119	0.9797 ± 0.0138	0.9885 ± 0.0075
DeepLab v3+	0.9867 ± 0.0060	10.8410 ± 4.0000	2.1777 ± 1.0120	0.9834 ± 0.0104	0.9901 ± 0.0103	0.9903 ± 0.0064
Ours	0.9869 ± 0.0067	10.7215 ± 4.0000	2.1107 ± 1.0312	0.9862 ± 0.0096	0.9878 ± 0.0124	0.9921 ± 0.0053

**Table 4:** Evaluation of the segmentation results for 300 tongue images from the SIPL-tongue dataset.

Method	DSC	HD (pixel)	MSD (pixel)	Precision	Sensitivity	Specificity
SegNet	0.9645 ± 0.0194	26.3156 ± 33.4805	5.9745 ± 6.3669	0.9429 ± 0.0382	0.9871 ± 0.0114	0.9831 ± 0.0118
FCN	0.9646 ± 0.0148	14.4019 ± 5.4493	4.4256 ± 1.6332	0.9466 ± 0.0324	0.9843 ± 0.0144	0.9843 ± 0.0144
PSPNet	0.9680 ± 0.0138	12.9473 ± 5.1630	4.0266 ± 1.6854	0.9519 ± 0.0298	0.9854 ± 0.0132	0.9854 ± 0.0132
DeepLab v3+	0.9699 ± 0.0148	12.5066 ± 6.2588	3.8472 ± 1.8831	0.9483 ± 0.0299	0.9931 ± 0.0060	0.9840 ± 0.0103
Ours	0.9726 ± 0.0136	11.2963 ± 5.7781	3.4737 ± 1.7573	0.9566 ± 0.0294	0.9897 ± 0.0085	0.9868 ± 0.0096

**Table 5:** Ablation study on the SIPL-tongue dataset.

Method	DSC	H D(pixel)	MSD (pixel)	Precision	Sensitivity	Specificity
Baseline	0.9611 ± 0.0141	17.0623 ± 13.9580	5.1126 ± 2.2745	0.9387 ± 0.0324	0.9856 ± 0.0146	0.9809 ± 0.0110
+RFB	0.9654 ± 0.0124	13.8587 ± 5.0418	4.3694 ± 1.5632	0.9485 ± 0.0279	0.9836 ± 0.0148	0.9841 ± 0.0095
+FPN	0.9689 ± 0.0128	13.2633 ± 7.6874	3.9478 ± 1.6488	0.9515 ± 0.0292	0.9878 ± 0.0116	0.9850 ± 0.0099
+MS	0.9726 ± 0.0136	11.2963 ± 5.7781	3.4737 ± 1.7573	0.9566 ± 0.0294	0.9897 ± 0.0085	0.9868 ± 0.0096

+: adding a new module or strategy based on the last row instead of the baseline. RFB: embedding RFB block into the segmentation network. FPN: employing the FPN structure. MS: fusing multiscale feature maps before final pixel prediction.

## Data Availability

The HIT-tongue dataset used in this research can be taken from https://github.com/BioHit/TongeImageDataset. The SIPL-tongue dataset used in this research has not been allowed to be public now because this dataset comes from an ongoing collaborative project and is being used for other research at the same time.
